# Complete Genome Sequence of a Virulent *Streptococcus agalactiae* Strain, 138P, Isolated from Diseased Nile Tilapia

**DOI:** 10.1128/genomeA.00295-14

**Published:** 2014-04-17

**Authors:** Julia W. Pridgeon, Dunhua Zhang

**Affiliations:** Aquatic Animal Health Research Unit, USDA, ARS, Auburn, Alabama, USA

## Abstract

*Streptococcus agalactiae* strain 138P was isolated from the kidney of diseased Nile tilapia in Idaho during a 2007 streptococcal disease outbreak. The full genome sequence of *S. agalactiae* 138P is 1,838,701 bp. The availability of this genome will allow comparative genomics analysis to identify genes for antigen discovery and vaccine development.

## GENOME ANNOUNCEMENT

*Streptococcus agalactiae* (group B streptococcus) is a Gram-positive pathogen causing human meningitis (1–3), neonatal sepsis (4), and pneumonia (5). In cattle, *S. agalactiae* is a major cause of bovine mastitis, a dominant health disorder affecting milk production (6, 7). In fish, *S. agalactiae* causes meningoencephalitis (8). In 2001, *S. agalactiae* was responsible for a massive fish kill at the Kuwait Bay, causing a loss of 2,500 metric tons of wild mullet (*Liza klunzingeri*) (9). Large-scale streptococcal outbreaks occurred frequently in tilapia farms of China from 2009 to 2011, involving >95% of farms, with a cumulative mortality of 30 to 80% (10–12). Reports from China indicate that the prevalent strains of *Streptococcus* causing disease in tilapia have shifted from *Streptococcus iniae* to *S. agalactiae* (10–12). In 2007, a streptococcal disease outbreak occurred in cultured Nile tilapia in Idaho. The strain 138P is a representative isolate collected from that disease outbreak.

The genome of *S. agalactiae* 138P was sequenced using the Illumina 1500 HiSeq platform. BioNumerics (Applied Maths) was used to *de novo* assemble a total of 30,870,348 sequence reads with an average length of 100 bp. The genomic contigs were used to search for sequence homology with deposited genome sequences in the GenBank nucleotide database using BLASTn. The first 6,000 bp of the genome sequence of *S. agalactiae* 138P shared 100% identities with the *S. agalactiae* strain 2-22 genome (accession no. FO393392). Therefore, strain 2-22 was used as a reference genome for this study. The whole genome of *S. agalactiae* 138P is 1,838,701 bp with a G+C content of 35.5%. The RAST server (13) predicted 1,891 coding sequences, including 305 involved in carbohydrate catabolism, 160 in protein metabolism, 141 in synthesis of amino acids and derivatives, 135 in cell wall and capsule synthesis, 103 in RNA metabolism, 101 in DNA metabolism, 95 in cofactors, 89 in nucleoside and nucleotide synthesis, 72 in fatty acid synthesis, 65 in membrane transport, 51 in virulence, 51 in stress response, 35 in phosphorus metabolism, 30 in cell division and cell cycle, 25 in regulation and cell signaling, 16 in iron acquisition and metabolism, 15 in potassium metabolism, and 3 in motility and chemotaxis. RAST also predicted 5 copies of 5S rRNA, 6 copies of 16S rRNA, and 6 copies of 23S rRNA.

Based on comparison with the genome of *S. agalactiae* 2-22, *S. agalactiae* 138P had the following three added features: (i) a 144-bp sequence between 524,842 nucleotides (nt) and 524,985 nt coding for a hypothetical protein, (ii) a 198-bp sequence between 1,006,860 nt and 1,007,057 nt coding for a transcriptional regulator belonging to the Cro/Cl family, and (iii) a 393-bp sequence between 1,009,336 nt and 1,008,944 nt coding for a hypothetical protein.

### Nucelotide sequence accession number.

The complete genome sequence of *S. agalactiae* 138P was deposited in GenBank under the accession no. CP007482.

## References

[1] Sanz-Rojas P Cabeza-Osorio L Hermosa C Serrano-Heranz R 2013 Acute meningitis by *Streptococcus agalactiae* in a immunocompetent male. Rev. Esp. Quimioter. 26:78–79 (In Spanish.) 23546471

[2] Reguera A González MÁ Sánchez A Aguayo C 2011 *Streptococcus agalactiae* meningitis in an immunocompetent male. Enferm. Infecc. Microbiol. Clin. 29:707–708 (In Spanish.) 10.1016/j.eimc.2011.05.020 21940070

[3] Martín Diaz RM Ruiz-Giardín JM Gonzalo Pascua S Canora Lebrato J 2010 Meningitis by *S*. *agalactiae* in a nonpregnant immunocompetent woman. Rev. Clín. Esp. 210:591–592 (In Spanish.) 10.1016/j.rce.2010.05.013 21036349

[4] Jawa G Hussain Z da Silva O 2013 Recurrent late-onset group B *Streptococcus* *sepsis* in a preterm infant acquired by expressed breastmilk transmission: a case report. Breastfeed. Med. 8:134–136 (In Spanish.) 10.1089/bfm.2012.0016 23373437

[5] Villena Ruiz MA Olalla Sierra J de la Torre Lima J García-Alegría J 2009 *Streptococcus agalactiae* induced cavitated pneumonia. Rev. Clín. Esp. 209:252–254. 10.1016/S0014-2565(09)71245-7 19480785

[6] Katholm J Rattenborg E 2010 The surveillance program of *Streptococcus agalactiae* in Danish dairy herds, p 1989–2008 *In* Hillerton J, (ed), Proceedings of the 5th IDF Mastitis Conference 2010 Mastitis Res. Into Practice. N Z Veterinary Association Vet. Learn. Christchurch, NZ

[7] Richards VP Lang P Bitar PD Lefébure T Schukken YH Zadoks RN Stanhope MJ 2011 Comparative genomics and the role of lateral gene transfer in the evolution of bovine adapted *Streptococcus agalactiae*. Infect. Genet. Evol. 11:1263–1275. 10.1016/j.meegid.2011.04.019 21536150 PMC3139733

[8] Liu G Zhang W Lu C 2012 Complete genome sequence of *Streptococcus agalactiae* GD201008-001, isolated in China from tilapia with meningoencephalitis. J. Bacteriol. 194:6653. 10.1128/JB.01788-12 23144401 PMC3497532

[9] Glibert PM Landsberg JH Evans JJ Al-Sarawi MA Faraj M Al-Jarallah MA Haywood A Ibrahem S Klesius P Powell C Shoemaker C 2002 A fish kill of massive proportion in Kuwait Bay, Arabian Gulf, 2001: the roles of bacterial disease, harmful algae, and eutrophication. Harmful Algae 1:215–231. 10.1016/S1568-9883(02)00013-6

[10] Chen M Wang R Li LP Liang WW Li J Huang Y Lei AY Huang WY Gan X 2012 Screening vaccine candidate strains against *Streptococcus agalactiae* of tilapia based on PFGE genotype. Vaccine 30:6088–6092. 10.1016/j.vaccine.2012.07.044 22867719

[11] Chen M Li LP Wang R Liang WW Huang Y Li J Lei AY Huang WY Gan X 2012 PCR detection and PFGE genotype analyses of streptococcal clinical isolates from tilapia in China. Vet. Microbiol. 159:526–530. 10.1016/j.vetmic.2012.04.035 22677479

[12] Ye X Li J Lu M Deng G Jiang X Tian Y Quan Y Jian Q 2011 Identification and molecular typing of *Streptococcus agalactiae* isolated from pond-cultured tilapia in China. Fish Sci. 77:623-632. 10.1007/s12562-011-0365-4

[13] Aziz RK Bartels D Best AA DeJongh M Disz T Edwards RA Formsma K Gerdes S Glass EM Kubal M Meyer F Olsen GJ Olson R Osterman AL Overbeek RA McNeil LK Paarmann D Paczian T Parrello B Pusch GD Reich C Stevens R Vassieva O Vonstein V Wilke A Zagnitko O 2008 The RAST Server: rapid annotations using subsystems technology. BMC Genomics 9:75. 10.1186/1471-2164-9-75 18261238 PMC2265698

